# Quantifying solvent action in oil paint using portable laser speckle imaging

**DOI:** 10.1038/s41598-020-67115-1

**Published:** 2020-06-29

**Authors:** Lambert Baij, Jesse Buijs, Joen J. Hermans, Laura Raven, Piet D. Iedema, Katrien Keune, Joris Sprakel

**Affiliations:** 1 University of Amsterdam, Van ‘t Hoff Institute for Molecular Sciences, PO box 94720, 1090GD Amsterdam, The Netherlands; 2 Rijksmuseum, Conservation and Science, PO box 74888, 1070DN Amsterdam, The Netherlands; 3 Wageningen University and Research, Department of Physical Chemistry and Soft Matter, Wageningen, The Netherlands

**Keywords:** Imaging techniques, Mechanical properties, Imaging studies

## Abstract

The exposure of oil paintings to organic solvents for varnish removal or to water for the removal of surface dirt can affect the chemical and physical properties of oil paint in an undesired way. Solvents can temporarily plasticise and swell the polymerised oil paint binding medium, enhancing both the thermal mobility and mechanical displacement of pigments embedded in this film. The enhancement of these microscopic motions can affect both the chemical and physical stability of the object as a whole. In order to minimise solvent exposure during cleaning, an analytical method that can quantitatively measure the microscopic motions induced by solvent uptake, is required first. In this study, we use Fourier Transform Laser Speckle Imaging (FT-LSI) and a newly developed portable FT-LSI setup as highly resolved motion detection instruments. We employ FT-LSI to probe pigment motion, with high spatiotemporal resolution, as a proxy for the destabilising effects of cleaning solvents. In this way, we can study solvent diffusion and evaporation rates and the total solvent retention time. In addition, qualitative spatial information on the spreading and homogeneity of the applied solvent is obtained. We study mobility in paint films caused by air humidity, spreading of solvents as a result of several cleaning methods and the protective capabilities of varnish. Our results show that FT-LSI is a powerful technique for the study of solvent penetration during oil paint cleaning and has a high potential for future use in the conservation studio.

## Introduction

Cleaning is considered to be an important step in the conservation of oil paintings^1,2^. However, it is known that the exposure of oil paints to organic solvents for varnish removal, or to water for the removal of surface dirt, can affect the physicochemical properties of the paint in an undesired way^3–5^. For example, exposure to organic solvents can lead to embrittlement of paint^6,7^ and to the extraction^8–14^ and redistribution^15^ of soluble paint components. An increased rate of formation of degradation products, such as crystalline metal soaps (complexes of metal ions and long-chain saturated fatty acids), has also been reported^15,16^. Although a considerable amount of progress has been made^3,4,17–19^, the knowledge of the influence of solvent-based cleaning on fundamental chemical processes in oil paint does not currently allow for a reliable assessment of the risks involved in cleaning. Besides effects induced by solvent exposure, it is known that factors such as relative humidity (RH)^20,21^, fluctuations in temperature^22^ and exposure to light^23^ can enhance the alteration and degradation of oil paints. However, it remains difficult to assess the importance of factors such as RH in terms of their influence on paint chemistry.

What exactly defines the impact of solvent action on paint is a complex interplay between many different chemical and physical phenomena. After the application of solvent, solvent swelling^24–26^, –diffusion^27^, –evaporation^28,29^, –leaching (extraction)^9–14^ and chemical reactions^15,16^ occur simultaneously. It is not possible to quantitatively measure the total physicochemical influence of these combined processes during cleaning practice, and conservators often assume that minimal exposure to water or solvents limits the risks associated with cleaning. However, minimising solvent exposure starts with identifying which processes, methods and solvents are least invasive and measuring these processes under realistic treatment conditions. This crucial question has remained largely unanswered because of the lack of analytical techniques that enable quantitative comparison of parameters that define the impact of solvent exposure on paint. The task of minimising solvent exposure during solvent-based cleaning requires a method that can quantitatively monitor the retention time and concentration of solvents reside inside oil paint. Ideally, such a technique should enable a quantitative comparison of cleaning methods or solvents on real paintings, should be portable and able to deal with heterogeneous paint surfaces. In addition, the instrumentation should feature an intuitive interface and should be easy to operate. Lastly but very importantly, data processing and interpretation should be fast (preferably on-the-fly) and should be easy to use for conservators. With such an analytical technique in hand, a conservator could then monitor how long a paint layer is exposed to a certain concentration of solvent and combine this with information on when the varnish removal is complete, thereby limiting the risks associated with solvent exposure to the paint layers.

In an attempt to classify solvents according to their diffusion coefficients, the authors have previously studied the diffusion of a range of solvents and water in linseed oil based ionomers and pigmented ZnO based paints using time-dependent attenuated total reflection Fourier transform infrared (ATR-FTIR) spectroscopy^27^. Important findings were that strongly swelling solvents generally diffuse faster than weakly swelling solvents and that all studied model systems showed similar diffusion behaviour, regardless of the presence of pigments^27^. While being good models for intact oil paint, these systems contain relatively pristine binding medium without (micro) cracks. Moreover, time-dependent ATR-FTIR spectroscopy does not allow the measurement of porous or brittle paints because it relies on constant and reproducible contact between the sample and the ATR crystal throughout the measurement. Another quantitative technique used to study solvent presence in paint is the NMR MOUSE (Mobile Universal Surface Explorer)^30^. Fife *et al*. compared the stiffness of two paintings from the same artist and time, one of which was never cleaned and one that had been repeatedly exposed to organic solvents^7^. It was shown that the painting that had undergone numerous solvent-based varnish removals was significantly stiffer throughout the depth of the painting. Angelova *et al*. employed the NMR MOUSE to study water ingress in acrylic emulsion paints^31^. However, due to the limited time-resolution of the NMR MOUSE, the uptake of rapidly diffusing solvents can not be monitored^29^. Moreover, the NMR-MOUSE does not provide spatially-resolved information which can assess the homogeneity of the result after the cleaning treatment.

In this study, we aim to develop a real-time, quantitative and non-invasive tool to probe the destabilising effects of solvents on paint surfaces. To do so, we use Fourier transform laser speckle imaging (FT-LSI) to probe the motion of pigment particles in the paint film. Enhanced motion of these pigments, due to plasticisation or swelling of their matrix, is a proxy for the presence, and effects, of solvents. This enables us to obtain spatially-resolved information on solvent penetration in oil paint. LSI is a light-scattering technique that was developed in the 1980s as a medical imaging tool to visualise subcutaneous blood flow^32^. A great advantage of LSI is that it does not require specific sample preparation and can be used non-invasively on any opaque surface. LSI has recently been applied to study the evolution of dynamics in drying paint^33,34^ and to capture the slow dynamics of drying artist oil paints and varnishes^35^. In the study by Pérez^35^, slow drying processes were monitored using discontinuous data collection at 10 fps every minute and data processing was performed after the raw speckle image collection. In this study, we use recently developed quantitative methods for on-the-fly data processing on a portable and low-cost (≈5 k euro) LSI setup^36^. With this setup we can collect up to 60 fps of raw speckle images continuously, allowing for the measurement of fast dynamics of solvent penetration during cleaning at a high frame-rate. Because organic solvents are transparent and do not scatter light, the measured LSI dynamics in oil paint observed after solvent exposure are the result of nanoscale motions of scattering pigment particles induced by solvent transport inside the paint. In our measurements, the nanoscale motions of scattering pigments are composed of two components: (1) thermal motion that probes the local mechanical (microrheological) properties of the paint and (2) convective motion induced by solvent molecules due to swelling or de-swelling of the paint matrix during solvent sorption or desorption. We investigate how transport phenomena, such as solvent diffusion and flow, are related to the measured LSI signal and to what extent either of these transport phenomena occur in our paint samples.

ZnO and linseed oil (ZnO-LO) based model systems are used to investigate solvent transport in oil paint models and to study how the rate of the investigated transport processes depends on paint degradation or environmental conditions. ZnO is widely used in oil paintings^37^ and associated to many types of degradation phenomena, most importantly the breakdown of ZnO itself^38^ and the formation of crystalline zinc soaps^16,39^. Linseed oil (LO) is widely used in oil paintings because it possesses excellent drying properties and consists of a mixture of triacylglycerides (TAGs) that mostly contain linolenic acid (C18:3), linoleic acid (C18:2), and oleic acid (C18:1) side chains. Upon drying and ageing, LO forms a tightly crosslinked polymer network. The network structure of the polymeric binding medium determines the rate and type of solvent transport in the paint. In this study, we perform experiments on lab-made model paint samples because this allows for full control over the sample composition, type of support, sample geometry and history of the samples.

To investigate the detection limit of the LSI setup for solvents inside paint, we set out to study complete swelling and de-swelling with ethanol, a solvent commonly used to dissolve aged natural varnishes on oil paintings. Subsequently, the effects of different relative humidity (RH) conditions in ZnO-LO films are measured and compared within a range of relative humidities recommended for museums^40^. Next, the relation between the rate and type of solvent transport and the paint degradation on a molecular level are investigated using both LSI and ATR-FTIR. Finally, different solvent application times and methods of solvent application are compared. The effects of varnishes are systematically studied using artificially aged dammar varnish with different thicknesses. A range of solvents that is typically used for varnish or surface dirt removal, as well as a selection of green solvents that have been suggested as new alternatives for some of these solvents, is compared.

Our preliminary experiments on aged model paint samples are expected to aid conservators in making informed decisions when choosing solvents or application methods. Ultimately, a portable FT-LSI setup with on-the-fly data processing could become a valuable analytical tool in the conservation studio by providing measurable criteria for the selection of solvents and cleaning methods during the initial phase of cleaning and solubility tests on real paintings.

## Results and Discussion

### De-swelling of a saturated paint film

The most extreme case of solvent transport in paint is complete swelling and de-swelling. The degree of equilibrium (maximum) swelling serves as an important parameter that determines the effects of solvents on oil paint and swelling has been extensively studied in the literature^24–27,41^.

**Figure 1:**
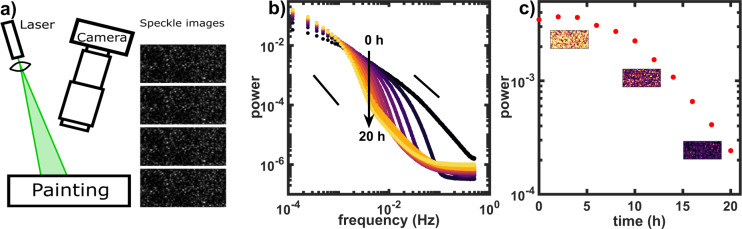
(**a**) Schematic representation of a typical FT-LSI experiment where the laser illuminates a painting, resulting in interfering back-scattered light. These characteristic speckle patterns are captured with a camera. Microscopic motion of light scattering pigments in the paint results in a change in the speckle pattern over time. The change in the speckle pattern over time is quantified using the Fourier transform, which provides a power spectrum for every pixel and results in a 4-dimensional dataset (x, y, time, frequency). (**b**) Power spectra of the de-swelling of a ZnO model system, showing the power of motion as a function of the measured frequency range from 0 hours (black) to 20 hours (light yellow) after the start of the de-swelling. Indicative slopes of −2 (left black line) for convective behaviour and −1.5 (right black line) for diffusive behaviour are indicated. (**c**) In depth analysis of the 0.0033 Hz frequency (arrow in b). We map the power of this frequency for every pixel to obtain a false-colour movie. The three snap-shots of the movie are presented and show a spatially homogeneous decrease of dynamics. By averaging every frame spatially we obtain the time-trace which gives a good overview of the whole experiment.

To determine the detection limit of the FT-LSI setup for solvent in oil paint, a ZnO-LO model system on glass support was saturated in ethanol for 2 hours to achieve complete swelling^27^, after which the de-swelling process was monitored using LSI. After removal from the ethanol bath, the paint was left to dry to the air in the FT-LSI set-up (Fig. 1a), which is described in the methods section. The speckle patterns were analysed in real-time using a recently established Fourier-transform algorithm. Fourier inversion of the temporal intensity fluctuations for each speckle, give access to a function, the power-spectral density, or power spectrum in short, that contains all relevant information about the type and rate of the dynamic processes that occur inside the paint film. The power spectrum for a specific dynamical process will show a low frequency plateau and a decay at frequencies higher than a characteristic decay frequency, where the slope (on a log-log representation) is indicative of the nature of the scatterer motion; with a slope = −1.5 for diffusive motion and −2 for convective motion. The characteristic decay frequency is a measure for the rate of the dynamics; slow diffusion leads to decay at high frequencies, while fast diffusion leads to decay at much lower frequencies. For a complete overview of the method we refer to Buijs *et al*.^36^.

During the de-swelling process, raw speckle images were collected over the course of 20 hours and analysed to obtain the power spectra that are shown in Fig. 1b. Ethanol is transparent to green light and therefore cannot be measured with FT-LSI. However, the presence of ethanol or other solvents will temporarily plasticise the chemically-crosslinked oil paint network, increasing the mobility of scatterers embedded therein, creating an acceleration of the temporal fluctuations in the scattered intensity that is detected with FT-LSI. Investigating Fig. 1b over the whole frequency range, it becomes clear that from t = 20 h to t = 0 h (increasing ethanol concentration), the power spectra and the inflection point shift to lower frequencies, which indicates that the effective diffusion coefficient increases with increasing ethanol concentration. The shape of the power spectra deviates from the characteristic shape described in the previous paragraph and reference^36^ because we measure a mixture of processes with different nature and characteristic timescales such as thermal motion, de-swelling and flow through micro-channels.

The value of the power spectral density at a given frequency, or power, can be used as a scalar proxy for the amplitude of dynamic processes, *e.g*. the relatively plasticity of the paint film. By following the power at a given frequency in time, temporal changes in the plasticity can be directly monitored. Figure 1c shows the power of the 0.0033 Hz frequency over the course of 20 hours. From this time-trace it is clear that, although the power has decreased by one order of magnitude, there still is a measurable change of dynamics going on after 20 hours. Using the theoretical description of (de)swelling of our paint models^27^, the swelling at t = 20 h is predicted to be <0.5% of the equilibrium swelling (at t = 0 h). It is thus clear that FT-LSI is able to measure motion that is induced by the presence of very small amounts of solvents inside the paint (Fig. 1c).

### Air humidity

Having established that FT-LSI is sensitive to low concentrations of solvents in paint, we investigated if subtle differences in environmental RH on the paint plasticity can be measured with LSI. The effects of environmental RH on oil paint are an important aspect in determining climate conditions in museums, which are often advised around 50% with limited fluctuations^40^. For oil paintings, it is known that high RH conditions can result in an increased rate of ester hydrolysis of TAGs in the oil binder and influence certain autoxidation pathways^38,42^. Ester hydrolysis can lead to the release of highly concentrated saturated fatty acids (SFAs) in an oil paint, thereby increasing the risks of (crystalline) metal soap formation when metal ions (for example derived from lead white (2PbCO_3_ Pb(OH)_2_) or zinc white (ZnO)) are present^16,43^. Even when the paint is already cured, high RH can also stimulate the formation of carboxylic acid groups^38^. The increased formation of carboxylic acid groups can indirectly lead to the formation of crystalline zinc soaps, because carboxylic acid groups stimulate the formation of amorphous zinc carboxylates^38^, which are intermediates in the formation of crystalline zinc soaps^16^.

**Figure 2:**
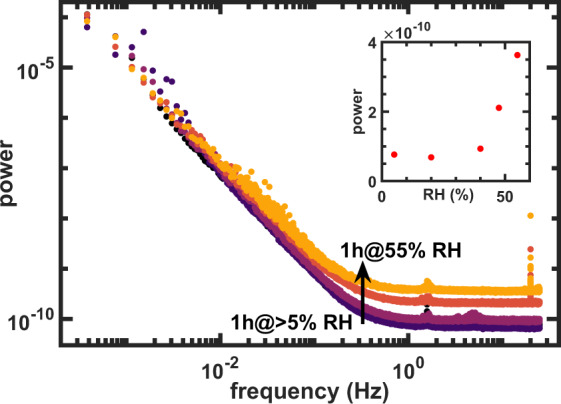
Power spectra of ZnO model painting with increasing humidity (dark to light yellow). Inset: mean power at high (>1 Hz) frequency. The dynamics increase significantly above 40% RH.

With the molecular mechanisms of paint degradation described above in mind, a ZnO model system was measured with LSI at different RH levels inside a climate box. For each humidity, the sample was left to equilibrate for 1 hour and then measured for 1 hour at room temperature (RT) after which the RH was increased for the next measurement cycle. The obtained power spectra are shown in Fig. 2 and the inset shows the average measured power at high frequencies (>1 Hz). A strong increase in dynamics at humidities above 40% was observed, which is the result of increased molecular motion in the paint. The fact that relatively small differences in RH conditions (40–50–55% RH, inset of Fig. 2) induce large changes in the dynamics inside oil paint, underlines the great potential of FT-LSI for monitoring the effects of solvents and water inside oil paint. Two explanations may contribute to the measured sudden increase in molecular motion around 40% RH: (1) moisture plasticises and swells the paint and (2) chemical paint alteration mechanisms, such as ester hydrolysis, could speed up significantly at this RH. It should be noted that it is currently unclear to what extent either of these two factors contribute to the measured power spectrum. Most likely, both these processes will go hand-in-hand. As such, our results may lead to re-evaluation of safe storage conditions for oil paintings. In the future, it could be worthwhile to investigate the relation between bulk viscoelasticity and RH with dynamic mechanical analysis (DMA). Since the glass transition temperature (*T*_*g*_) of ZnO-LO is close to RT^27^, it may be that subtle differences in RH induce a transition to the rubbery regime, providing further explanation for the strong increase in molecular motions.

### Effects of ester hydrolysis on solvent transport

Due to the wide variety in the composition of paint materials and the conditions during the drying and storage of paintings, a wide variety of different porosities and network structures is found in paintings. Differences in porosity are expected to have a strong effect on the rate of solvent transport in paint and the retention of solvents inside paints. To describe solvent transport, a distinction is often made between convective solvent transport in micro-channels and solvent diffusion in the inherent free volume of the polymer network^44^. Convective transport in paint is generally several orders of magnitude faster that diffusion^45^. In our experiments, a range of processes consisting of solvent sorption, desorption and evaporation contribute to the total FT-LSI signal intensity and decay rate that is measured. The rate of FT-LSI signal decay thus gives information on sorption, desorption and evaporation combined. Although the extent to which sorption, desorption and evaporation contribute to the measured signal is currently unknown, these processes combined define the *retention time*: the time solvents are retained in the paint layer. Knowing the solvent retention time and the rate of signal decay can help to decrease the risks associated with solvent-based cleaning because these important parameters determine how long the paint is plasticised by solvents. To investigate if LSI can monitor such differences, we studied solvent transport in oil paints that were subjected to prolonged exposure to very high RH conditions.

**Figure 3:**
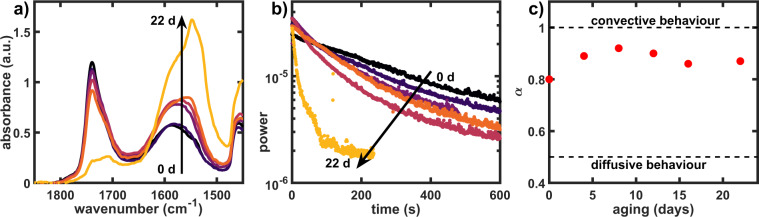
(**a**) ATR-FTIR spectra normalised on the CH_2_ stretching vibration (2929 cm^−1^) and (**b**) FT-LSI signal decay (1.6 Hz) for series ZnO-LO-0d–22d series where the series are coloured increasingly dark from 0d to 22d following the trend of the arrow. (**c**) Stretching exponent *α* where *α* = 0.5 corresponds to purely diffusive motion and *α* = 1 corresponds to purely convective behaviour. ROI for FT-LSI-signal integration were chosen as described in Fig. S3. The fitting procedure to obtain *α* is shown in Fig. S5.

A series of ZnO-LO paints that were subjected to accelerated ageing at 97% RH at 60 °C for 4 to 22 days (designated ZnO-LO-0d to ZnO-LO-22d) were used for these experiments. Because ZnO-based model paints easily hydrolyse in high RH conditions, the effects of ester hydrolysis on solvent retention and transport can be studied. Upon ageing, an increasingly matte appearance and extensive yellowing was observed. At the same time, hydrolysis of the esters in linseed oil, the liberation of free fatty acids (FAs) and, eventually, the formation of zinc soaps took place. Figure 3a shows a collection of ATR-FTIR spectra, clearly showing the increasing concentration of amorphous zinc carboxylates^38,46,47^ (COOZn, broad band at 1585 cm^−1^ in Fig. 3a) and ultimately, the formation of crystalline zinc soaps after 22 days of ageing (sharp peak at 1540 cm^−1^ in Fig. 3a).

**Figure 4:**
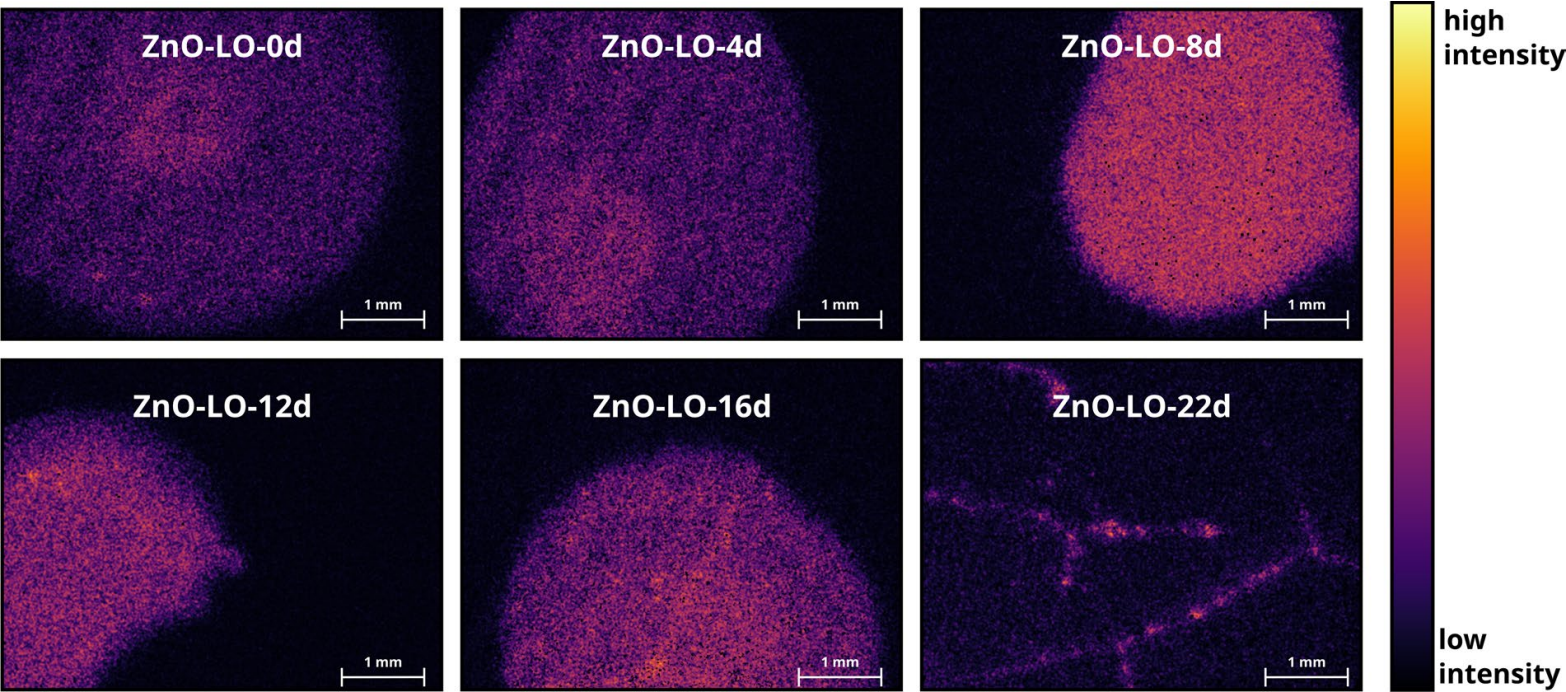
FT-LSI images (1.6 Hz) for ZnO-LO-0d–22d one minute after ethanol application (3 *μ*L droplet). Increasing solvent spreading and micro-crack formation upon ageing at 97% RH and 60 °C for 4 to 22 days. Solvent spreading and penetration rate correlates with increasing COOZn concentration upon ageing (ZnO-LO-0d–16d) and finally significant ester hydrolysis and zinc soap formation in ZnO-LO-22d.

Solvent retention was measured by placing a 3 *μ*L droplet of ethanol on top of the sample, waiting for the appearance of a dry surface (*t*_0_, as seen in the images by the droplet shrinking) and subsequent integration of the total intensity of the FT-LSI signal in a Region of interest (ROI) in the centre of the droplet. Supplementary movie ZnO-LO-22d.avi shows the raw speckle images obtained during the ZnO-LO-22d measurement side-by-side with the analysed FT-LSI movie. The FT-LSI signal decay for the ZnO-LO-0d–22d series is displayed in Fig. 3b, showing a significant increase in signal decay rate with increased ageing. In this series, the 1.6 Hz frequency was chosen in order to detect the fast changes in dynamics encountered during solvent exposure. Besides the significant increase in signal decay rate upon ageing, major qualitative differences in the FT-LSI images were observed in the strongly aged sample (see 22 days aged sample in Fig. 4). Especially ZnO-LO-22d clearly shows the appearance of micro-channels after solvent application.

The increased amount of crack formation in aged samples may explain the increased transport in lateral direction (Fig. 4, ZnO-LO-22d), and therefore the increased rate of solvent uptake. It has been shown in the literature that the rate of solvent sorption (*k*_*S*_) in oil paint is faster than desorption (*k*_*D*_) and the two rates are related by *k*_*D*_/*k*_*S*_ ≈ 0.65 in most cases^24^. If there is no significant interaction between solvent and paint that depends on the ageing, solvent desorption will also be faster in films that have been more severely aged. The combined faster spreading of the solvent due to transport in lateral direction and the faster desorption due to faster evaporation may explain the increased decay rate for samples with increased ageing, finally resulting in lower solvent retention in aged samples.

To investigate the type of solvent transport in more detail, the stretching exponent *α* was calculated (Fig. 3c). The value of *α* gives information about the nature of the measured motion: if *α* = 0.5, the motion is purely diffusive and if *α* = 1, the motion is purely convective^34^. The *α* factor was computed by fitting the slope of the power spectrum with a straight line where the slope is equal to −(1 + *α*) on a log-log axis^36^. The motion of pigment particles probed by LSI are the result of both the microrheological properties of the paint and the convective motion of the swelling. Figure 3c indicates that the pigment motion is partly diffusive and partly convective over the whole ageing series and does not show a significant change in *α* upon ageing. The solvent itself will have flow-like behaviour through micro-channels and cracks, but does not translate this motion directly to the pigment particles and therefore this is not noticeable in the computed *α*-values. In Supplementary Movie ZnO-LO-22d.avi it is visible that the solvent spreads out over a large area through cracks in the ZnO-LO-22d while Supplementary Movie ZnO-LO-0d.avi shows that the solvent remains localised in the area of the droplet in the ZnO-LO-0d sample. However, the mechanisms behind the plasticisation and swelling do not change. Consequently, a larger area is affected by the solvent in the aged films for a shorter period of time.

We conclude that there is a correlation between solvent retention (Figs. 3b and 4) and the degree of ageing measured by increased ester hydrolysis (Fig. 3a): increasingly aged samples show faster FT-LSI signal decay and more solvent spreading. Because the solvent is transferred out of the ROI that was used for integration of the LSI signal, it remains difficult to say to what extent this faster signal decay originates from either evaporation or convective transfer. In any case, our measurements confirm that an increase in crack formation results in an increase in both the rate of evaporation and the rate of solvent transport. As a result, the area of the paint that is exposed to solvents is significantly increased in aged samples, even when solvents are applied locally.

### Effects of solvent exposure time and varnishes

**Figure 5:**
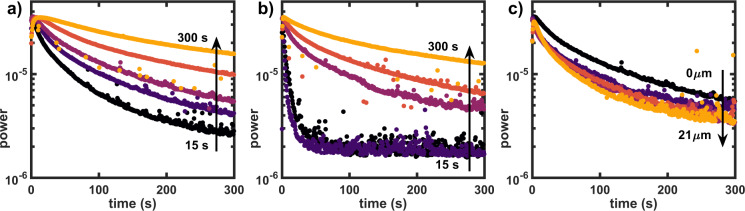
FT-LSI signal decay (1.6 Hz) time-series: 15–300 seconds Evolon-51% ethanol exposure, (**a**) unvarnished, (**b**) varnished (<7 *μ*m) and (**c**) varnish layers with increasing thickness ranging from <7–21 *μ*m and constant exposure time of 60 s. ROI for FT-LSI-signal integration were chosen as described in Fig. S3.

The effects of solvent exposure time and the presence of varnish on the solvent delivery inside the paint layer were investigated using Evolon CR tissue with a 51% loading of ethanol (Evolon-51%, see section comparing application methods)^48^. This cleaning method is known for it’s effectiveness in varnish removal and features an excellent reproducibility (see Fig. S1). The FT-LSI signal decay was measured for a series of 15–300 seconds exposure of Evolon-51% ethanol on an unvarnished ZnO-LO model system. The results are displayed in Fig. 5a, showing that the rate of FT-LSI signal decay strongly decreases with increasing exposure time. As expected, longer application times result in increased solvent delivery in the paint, as shown in Fig. 5a.

In a control experiment testing solvent-swollen varnish on glass, we investigated if varnish leftovers (without scattering pigments) can significantly contribute to the measured LSI signal. It was found that solvent-swollen varnish is an important contribution on short timescales (see Fig. S2) due to the rapidly changing refractive index of the varnish when swollen with solvent. As a result, interpreting the relative contribution of scattering inside the paint and scattering inside the varnish in measurements where varnish is left on the paint surface after solvent exposure, is difficult. To test if varnish removal was complete, a portable UV lamp was used to judge if varnish fluorescence was absent after all measurements. This method is also routinely used by conservators to determine if varnish removal is complete.

Figure 5b shows the effect of a thin (9 *μ*m) varnish layer. Judging by the similar signal decay rates obtained for 15 and 30 s exposure time, the solvent does not reach the paint layer in the first 30 s and the FT-LSI signal is dominated by the quickly decaying signal of swollen varnish. The incomplete removal of varnish after 15 and 30 s was confirmed by the presence of UV fluorescence, indicating that the relatively intense dynamics in the first seconds after solvent application may be partly explained by the signal of swollen varnish in the measurements using 15 and 30 s exposure time. Exposure times of 60 s or more yield similar results to the unvarnished samples, indicating that the varnish layer was indeed removed and unhindered solvent transport into the paint layer was possible after ≈60 s.

To explore the effects of the varnish thickness on the relative amount of solvent delivered to the paint in more detail, a series of varnished model systems with and increasing thickness of <7–21 ±2 *μ*m was prepared (thickness determined by optical coherence tomography^49^, OCT, see Fig. S4). In this series, the solvent exposure time was kept constant at 60 s. The results are displayed in Fig. 5c, clearly confirming that the presence of thicker varnishes results in smaller amounts of solvent penetrating into the paint layer. However, this effect is subtle compared to the exposure time series because doubling the varnish thickness did not double the protective duration.

### Comparison of solvents

Conservators use a wide range of different (mixtures of) organic solvents to dissolve and remove discoloured natural resin varnishes. The composition of these varnishes determines the choice of solvents for their effective removal, keeping the minimal influence to the underlying paint layers in mind. Like polymerised drying oils, varnishes generally increase in polarity upon ageing by the increasing formation of oxidised products. The increased polarity of the varnish can change its solubility in organic solvents significantly and require increasingly polar solvents to dissolve aged varnishes.

To investigate if FT-LSI can discriminate between the activity of different solvents, a series of solvents used for the cleaning of paintings was tested on an unvarnished paint surface. It is known that solvents can have vastly different diffusion rates, with water being among the slowest diffusing solvents and acetone one of the quickest^24,27,41,50^. During conservation cleaning practice, solvent diffusion- and evaporation rate are important because these combined factors determine how long solvents are present in a paint and how far they diffuse inwards. We used FT-LSI to measure this combined effect of diffusion and evaporation of acetone, ethanol, isopropanol, dimethyl carbonate (DMC), ethyl lactate (EL), *γ*–valerolactone (*γ*–VL) and *n*-hexane. Acetone, ethanol, isopropanol and hexanes are widely used in conservation studios, whereas DMC, EL and *γ*–VL have recently been suggested as green alternatives for varnish removal^29,51^.

FT-LSI probes changes in the dynamics of scattering pigments inside the paint film and therefore the power of the FT-LSI signal is not easily converted into a solvent concentration. The pigment motion is governed by the local visco-elasticity of the paint film, which is influenced by the presence of varying amounts of solvent in a complex way that is currently unknown. In principle, a thorough calibration of the solvent content on the scatterer motion would enable such a conversion but would need to be performed for each different type of matrix and solvent as it is sensitive to chemical details. However, for initial monitoring of solvent presence during artwork cleaning, such a conversion is not required.

**Figure 6:**
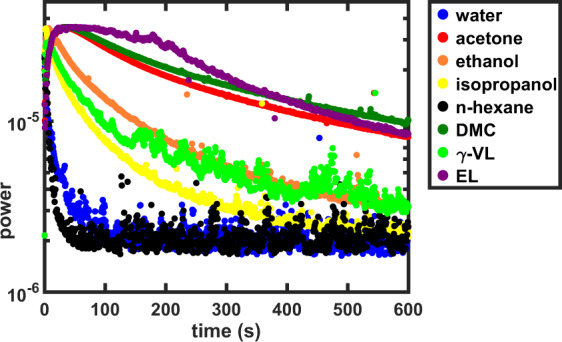
FT-LSI signal decay (1.6 Hz) for a series of solvents applied as a 20 *μ*L drop, left on the surface for 60 s and dried with filter paper. ROI for FT-LSI-signal integration were chosen as described in Fig. S3.

The FT-LSI signal decay of the 1.6 Hz frequency is displayed for a series of solvents in Fig. 6. The solvents were applied as a 20 *μ*L droplet, left on the surface for 60 s and carefully dried with filter paper. Although the translation of the FT-LSI signal into diffusion rates is not straightforward, it is interesting to observe that the trends in FT-LSI signal decay displayed in Fig. 6 are in agreement with known trends in diffusion rates: acetone > ethanol > isopropanol > water ≈ hexane^27^. The signal decay rates of EL and DMC are comparable and close to acetone, both are much faster than *γ*–VL. This effect can be partly explained by the fact that ethanol and acetone are much faster diffusing solvents than hexane and water^27^, resulting in further solvent penetration and longer plasticisation.

It should be noted that the FT-LSI signal does not necessarily correlate with the rate of varnish dissolution or leaching of soluble paint components, since these processes are governed by the solubility of these components in a given solvent. For example, although the FT-LSI signal decay rate for acetone, DMC and EL are highly similar, their rate of varnish dissolution on aged dammar varnish is vastly different, with acetone being faster than DMC and both acetone and DMC much faster than EL. The rate of leaching of soluble paint components is an important factor in cleaning studies but can not be measured by LSI. However, if solvent penetration in the paint is limited, it should be safe to assume that leaching is also minimised.

### Comparison of solvent application methods for varnish removal

Besides flexibility in the choice of solvents, a conservator can utilise a variety of methods to apply the solvents. Most of these methods have been developed to minimise the amount of solvent exposure and mechanical action on the surface and to increase the reproducibility of the cleaning action, resulting in a more homogeneously cleaned surface. We have used the portable FT-LSI setup^36^ to compare four methods of solvent application for varnish removal from ZnO-LO model paints, using ethanol as a solvent in all cases:

- The cotton swab, traditionally widely used for varnish removal
- Evolon CR tissue, an alternative used for varnish removal and composed of a Nylon/polyethylene terephthalate (PET) fabric^48^. The Evolon tissue can be loaded with different amount of solvent by equilibrating known amounts of tissue and solvent in a sealed container overnight. In our tests, the Evolon tissue was always covered with a thin sheet of Mylar (biaxially-oriented PET) during application.
- Nanorestore Max Dry (MD) gel, can be used for varnish or surface dirt removal and consists of semi-interpenetrating polyhydroxyethylmethacrylate (pHEMA) and polyvinylpyrrolidone (PVP) networks^52^.
- the ‘SRAL method’, employing the spreadable hydroxypropylcellulose (HPC, marketed as Klucel G) gel loaded with cleaning solvent on an impregnation tissue, removing the gel with an absorbing tissue and subsequent rinsing with a lower polarity solvent and a cotton swab^53^. Isopropanol was chosen for rinsing.

**Figure 7:**
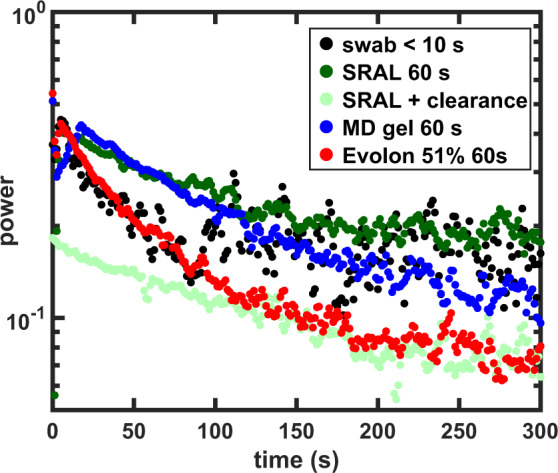
FT-LSI signal decay (1.6 Hz) for ethanol applied using the cotton swab, Evolon tissue, MD gel and SRAL method measured using the portable LSI setup. Except for the swab, the surface was always exposed to ethanol for 60 s, the swab was used until a satisfying result was obtained (<10 s). The dotted line in the Evolon image marks the edge of the evolon tissue. For comparison, the SRAL method was measured with and without isopropanol rinsing. The initial varnish thickness was 9 *μ*m. ROI for signal integration as described in Fig. S3.

**Figure 8:**
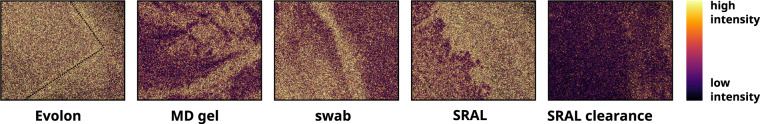
Scaled FT-LSI images obtained using the portable LSI setup. The images were taken directly after the simulated treatment with ethanol applied using the cotton swab, Evolon tissue, MD gel and SRAL method. Except for the swab, the surface was always exposed to ethanol for 60 s, the swab was used until a satisfying result was obtained (<10 s). The images from the SRAL method are shown with and without isopropanol rinsing. The initial varnish thickness was 9 *μ*m. The total area that was exposed to Evolon tissue was 1 × 1 cm, the dashed lines approximately indicate the boundaries where Evolon tissue was applied.

The resulting comparison of different methods of solvent application measured on the portable LSI setup is displayed in Fig. 7. Although the signal decay rates shown in Fig. 7 are not pure diffusion coefficients, the relative signal decay rates can be used as a measure of the amount of ethanol delivered into the paint. In this set of measurements, the absolute signal intensity is much higher compared to the lab-based LSI. These differences are not relevant for the comparison of different methods of solvent application within this data set. Figure 7 shows that the swab and Evolon feature a similar and relatively fast decay rate. Keeping in mind that the swab method was used until a satisfying varnish removal was obtained (<10 s), the dynamics are initially quite intense. The fast decay to a low intensity indicates that the amount of solvent delivered deeper into the paint by the swab and Evolon is small. This result is not in contradiction with our earlier result showing that the swab extracts more free FAs from deeper paint layers compared to Evolon or MD gel, because in that study all methods were compared at the same exposure time^15^. It should be noted that the time required to completely remove the varnish with Evolon was <60 s, implying that even shorter contact times could be used with this method. The initial signal intensity for the SRAL method (excluding clearance) and for the MD gel are much higher than the other methods, probably because a significant amount of varnish remained on the surface (see also Fig. 8) after the treatment. If varnish is left on the surface, the evaporation of solvent is hindered, explaining the relatively slow decay rate and high signal intensity even after 300 s. If the SRAL method is directly cleared with a swab afterwards, the signal intensity is very low due to the use of isopropanol, a slowly diffusing solvent (see Fig. 6).

A valuable addition to the quantitative information obtained from the portable FT-LSI signal integration is obtained from the LSI images displayed in Fig. 8, where the homogeneity of the solvent application can be compared qualitatively. Figure 8 shows that the Evolon tissue results in the most homogeneous application of the solvent. Diffusion of ethanol vapour outside the regions of the Evolon tissue (marked with a dotted line) is also observed, likely because the Evolon tissue is covered with a sheet of Mylar during application. Ethanol diffusion outside the regions of application may explain the formation of tidelines (unwanted regions were varnish is deposited outside the area of solvent application) which is frequently noted by conservators. It is often claimed that rigid gels such as the MD gel allow a more precise application of the solvent. Interestingly, this is not actually the case judging from the portable FT-LSI image for the MD gel. The relative heterogeneity visible in the image of the MD gel can be explained by the fact that the MD gel did not completely remove the varnish within 60 s, but rather redistributed part of the varnish over the area of application. For a fair comparison, the swab method was not used for 60 s but until a satisfying result was obtained (<10 s, as judged by the conservator using a portable UV lamp). Although the initial FT-LSI signal intensity is similar to the other methods, the varnish removal is very quick. A downside of the swab methods is also evident, showing that swabbing results in certain areas receiving more pressure and thus more solvent than others, judging from the heterogeneity that is visible in Fig. 8. The portable FT-LSI images show a heterogeneous solvent application by the SRAL method, but after clearance with isopropanol, the activity is homogeneous as well as of lower intensity than other methods.

Despite the higher noise levels compared to the lab-based setup, the data from the portable FT-LSI setup (Figs. 7 and 8) clearly shows quantitatively different results for different solvent application methods. Most importantly, the FT-LSI images (Fig. 8) are computed and displayed real-time during the measurement, immediately showing the qualitative differences and making the portable FT-LSI a valuable tool for conservators.

## Conclusions

LSI is a powerful and sensitive technique to study the motion of scattering pigments inside oil paint, showing the presence of solvent during solvent cleaning real-time. LSI detects the presence of ethanol in oil paint for more than 20 hours after saturation with solvent. In a set of measurements with increasing relative humidity, a strong increase in dynamics at humidities above 40% was observed, underlining the great sensitivity of LSI for detection low concentrations of solvents inside oil paints.

Increasingly aged paints showed solvent spreading over a larger area but a shorter overall solvent retention. A correlation between increased ester hydrolysis of the binding medium and rapid solvent flow in micro-cracks could be made. The effects of increasing solvent exposure time using Evolon tissue on varnished and unvarnished paints were studied. Increasing exposure times were shown to result in slower signal decay, indicating that more solvent is delivered inside the paint. Thin varnish layers protected the paint temporarily from solvent sorption, showing how long solvent exposure can be with minimal solvent penetration into the paint. LSI provides quantitative and qualitative spatial information on cleaning methods, which are required for a reliable risk assessment of application times or -methods. Important quantitative differences in the LSI signal decay rate and intensity could be identified for different solvents and solvent application methods. Moreover, we obtained qualitative spatial information regarding the heterogeneity of solvent application for different cleaning methods. Because LSI is an affordable, portable and non-invasive technique that provides real-time results, it can be a powerful asset in the conservation studio during initial cleaning and solubility tests.

Future work could be directed at comparing different solvent (mixtures) at different application times to find an optimum between varnish solubility and the amount of solvent delivered to the paint. An important future challenge in the development of LSI for cleaning tests on large paintings is the reduction of background vibrations of the canvas or panel during the testing procedure. Ideally, LSI could then be used to develop a standardised cleaning test procedure to tailor cleaning strategies to the unique properties of a given painting.

## Methods and Materials

### Lab-scale FT-LSI

LSI measurements were performed on a home-built set-up that was described previously^34^. Surfaces were illuminated by a 532 nm laser (Cobolt Samba, 1 W) which was expanded with a Galilean beam expander. The backscattered light was captured by a zoom lens (Qioptiq) and the speckle patterns are captured with a camera (Stemmer, Dalsa Genie). The speckle images were saved at high frame-rates (up to 200 fps) and analysed later with Fourier analysis. The maximum laser power used in experiments on the FT-LSI was 10 mW. Since the beam was expanded to an area with a diameter of >1 cm, the light intensity at the sample was about 0.13 mW/mm^2^. This instrument was used for all LSI experiments, except when specified otherwise.

### Portable FT-LSI

The other LSI experiments were performed on a portable FT-LSI set-up that was described in^36^ and can be used in the conservation studio. Surfaces were illuminated by a 532 nm laser (Cobolt Samba, 20 mW) which was expanded by a single bi-concave lens. The backscattered light was captured by a variable zoom objective (Navitar) and the speckle patterns were captured with a camera (Thorlabs, DCC3240N). The speckle images were collected at medium frame-rates (50 fps) and Fourier analysis is performed in real time on a tablet. The maximum laser power used in experiments on the portable FT-LSI was 5 mW. Since the beam was expanded to an area with a diameter of >1 cm, the light intensity at the sample was about 0.064 mW/mm^2^. The total cost of the hardware needed to construct the portable FT-LSI setup is around 5k euro.

### Fourier analysis

The FT analysis was performed as we described previously^36^. We start by computing the power spectral density. We perform the FFT on a time sequence of N speckle patterns, where N is a power of 2 for computational efficiency. Each (xyt)-voxel is treated as an intensity (I) time series of length N; all voxels are transformed simultaneously as a three dimensional matrix. First, we subtract the mean intensity for each voxel, to represent the time trace as intensity fluctuations around a mean of zero. This matrix is transformed to the frequency domain with a standard fast Fourier transform (fft) routine. Amplitudes corresponding to negative frequencies are discarded to obtain the single-sided amplitude spectrum, which is squared to obtain the power. The power is normalized by dividing by the frame rate and the trace lenght N, as well as dividing by <*I*^2^>−<*I*>^2^. The result is a three dimensional power matrix P(x;y;w) containing power spectral densities for each xy pixel. The frequencies w are equally spaced between 0 and (π* frame rate), with N = 2 + 1 unique frequencies. This matrix is either averaged spatially to obtain an averaged power spectrum, or the result of a single frequency is mapped to a surface to obtain a FT-LSI image for a particular frequency. On the lab scale FT-LSI all speckle images are first saved and FT analysis is performed later. On the portable FT-LSI the FT analysis is performed in real time on a stream of images.

### Sample preparation

Sample preparation was performed as we described previously^38^. Model paint samples containing ZnO (Sigma Aldrich, ≥99%) were made by grinding the pigments with cold-pressed untreated linseed oil (Kremer pigmente) in a 1:1 (w/w) ratio to a smooth paste with mortar and pestle. The Pigment Volume Concentration (PVC) was 19% in all samples. The mixture was applied to 50 × 75 mm glass slides and spread with a draw-down bar to achieve a wet thickness of 190 *μ*m. The samples were cured in the dark in air at 60 °C for 0–22 days at 97% RH, resulting in a dry thickness of about 150 *μ*m. Humidity was controlled using a saturated K_2_SO_4_ solution (for 97% RH) in a closed container and was determined using a Rotronic HL-1D temperature and humidity data logger. For ATR-FTIR analysis, 5 × 5 mm squares of the films were cut and lifted off the glass. Samples were varnished using a brush with a dammar solution in shellsol A and subsequently aged for 7 day under UV-A and UV-B radiation. The total radiation dosage was 1.4 × 10^7^ J/cm^2^ (UV-A) and 5.2 × 10^7^ J/cm^2^ (UV-B). Cross-sections of varnished model systems were embedded in Technovit 2000 LC resin and cured in a Technovit 2000 LC Technotray POWER - Light Polymerization Unit for for 30 min. The cross-section was sanded down using a MOPAS XS Polisher and wet and dry (Micromesh) polishing techniques.

### Simulated cleaning test procedure

The simulated cleaning test procedures were performed as we described previously^15^. Nanorestore Max Dry (MD) was used as received from CSGI (www.csgi.unifi.it). Gels were kept in a sealed container loaded with ethanol for at least 12 hour before use and dried with paper tissue before application. Evolon CR tissue (www.deffner-johann.de/evolonr-cr.html) was cut into 1 × 1 cm squares, washed with acetone and ethanol using a Buchner funnel, dried and subsequently loaded with ethanol. The Evolon samples with controlled loading were kept overnight in a sealed container loaded with ethanol. Strips of 2 × 5 cm were loaded with 51% (92.3 mg Evolon/149.1 mg ethanol) before use. During solvent application, Evolon and gel samples were covered with a Mylar (biaxially-oriented polyethylene terephthalate) film to avoid solvent evaporation from the top. Hand-rolled cotton swabs were used and swabbing was carried out by a trained conservator. Swab cleaning was always carried out by Laura Raven, Rijksmuseum.

### ATR-FTIR spectroscopy

ATR-FTIR spectroscopy was performed as we described previously^38^. ATR-FTIR spectra were measured on a Perkin-Elmer Frontier FT-IR spectrometer fitted with a Pike GladiATR module and a diamond ATR-crystal. Spectra were averaged over 4 scans. To integrate overlapping absorption bands, automated data correction and integration algorithms were written using Wolfram Mathematica software.

### OCT

OCT measurements of varnish thickness were performed on a Thorlabs Telesto PS OCT with a central wavelength of 1300 nm. This setup features a max depth of 3.75 mm in air, a depth resolution of 5.5 *μ*m in air and 3.7 *μ*m at a refractive index of 1.5. The lateral resolution (beam diameter) was 13 *μ*m, the field of view 10 × 10 mm and the working distance 2.5 cm.

## Supplementary information

Supplementary information

Supplementary movie ZnOLO-0d

Supplementary movie ZnO-LO-4d

Supplementary movie ZnO-LO-8d

Supplementary movie ZnO-LO-12d

Supplementary movie ZnO-LO-16d

Supplementary movie ZnO-LO-22d

.tex file

## References

[1] Ruhemann, H. & Plesters, J. *The cleaning of paintings; problems and potentialities;* (Faber, London, 1968).

[2] Stoner, J. H. & Rushfield, R. A. *The conservation of easel paintings* (Routledge London, 2012).

[3] Phenix A, Sutherland K (2001). The cleaning of paintings: effects of organic solvents on oil paint films. Studies in Conservation.

[4] Mecklenburg MF, Charola AE, Koestler RJ (2013). New Insights into the Cleaning of Paintings: Proceedings from the Cleaning 2010 International Conference, Universidad Politécnica de Valencia and Museum Conservation Institute. Smithsonian Contributions to Museum Conservation.

[5] Baij, L., Hermans, J., Ormsby, B., Noble, P., Iedema, P., & Keune, K. (2020). A review of solvent action on oil paint. *Heritage Science*, **8(1)**, 43, https://doi.org/10.1186/s40494-020-00388-x

[6] Hedley G, Odlyha M, Burnstock A, Tillinghast J, Husband C (1990). A study of the mechanical and surface properties of oil paint films treated with organic solvents and water. Studies in Conservation.

[7] Fife GR (2015). Characterization of aging and solvent treatments of painted surfaces using single-sided NMR. Magnetic Resonance in Chemistry.

[8] Erhardt D, Tsang J-S (1990). The extractable components of oil paint films. Studies in Conservation.

[9] van den Berg JD, van den Berg KJ, Boon JJ (2002). Identification of non-cross-linked compounds in methanolic extracts of cured and aged linseed oil-based paint films using gas chromatography-mass spectrometry. Journal of Chromatography A.

[10] Sutherland K (2003). Solvent-Extractable Components of Linseed Oil Paint Films. Studies in conservation.

[11] Spyros A, Anglos D (2004). Study of aging in oil paintings by 1D and 2D NMR spectroscopy. Analytical Chemistry.

[12] Spyros A, Anglos D (2006). Studies of organic paint binders by NMR spectroscopy. Applied Physics A: Materials Science and Processing.

[13] Zumbuhl S, Scherrer NC, Engel NL, Muller W (2014). The kinetics of dissolution of varnishes: The influence of vapour pressure on the rate of solvent action. ICOM-CC, 17th Triennial Conference, Chipperfield.

[14] Casoli A, Di Diego Z, Isca C (2014). Cleaning painted surfaces: evaluation of leaching phenomenon induced by solvents applied for the removal of gel residues. Environmental Science and Pollution Research.

[15] Baij L (2019). Solvent-mediated extraction of fatty acids in bilayer oil paint models: a comparative analysis of solvent application methods. Heritage Science.

[16] Baij L, Hermans JJ, Keune K, Iedema P (2018). Time-Dependent ATR-FTIR Spectroscopic Studies on Fatty Acid Diffusion and the Formation of Metal Soaps in Oil Paint Model Systems. Angewandte Chemie International Edition.

[17] Kahrim K (2009). The application of *in situ* mid-FTIR fibre-optic reflectance spectroscopy and GC-MS analysis to monitor and evaluate painting cleaning. Spectrochimica Acta - Part A: Molecular and Biomolecular Spectroscopy.

[18] Baglioni, P., Baglioni, M., Bonelli, N., Chelazzi, D. & Giorgi, R. Smart Soft Nanomaterials for Cleaning. In *Nanotechnologies and Nanomaterials for Diagnostic, Conservation and Restoration of Cultural Heritage*, 171–204, 10.1016/B978-0-12-813910-3.00009-4 (Elsevier, Dordrecht, 2019).

[19] Angelova, L. V., Ormsby, B., Townsend, J. & Wolbers, R. (eds.) *Gels in the conservation of art* (Archetype Publications, London, 2018).

[20] Foster GM, Ritchie S, Lowe C (2003). Controlled temperature and relative humidity dynamic mechanical analysis of paint films. Journal of Thermal Analysis and Calorimetry.

[21] Ormsby B, Foster G, Learner T, Ritchie S, Schilling M (2007). Improved controlled relative humidity dynamic mechanical analysis of artists’ acrylic emulsion paints: Part II. General properties and accelerated ageing. Journal of Thermal Analysis and Calorimetry.

[22] Michalski, S. Paintings–Their Response to Temperature, Relative Humidity, Shock, and Vibration. *Art in Transit: Studies in the Transport of Paintings* 223–248 (1991).

[23] Monico L (2011). Degradation Process of Lead Chromate in Paintings by Vincent van Gogh Studied by Means of Synchrotron X-ray Spectromicroscopy and Related Methods. 1. Artificially Aged Model Samples. Analytical Chemistry.

[24] Stolow N (1957). The action of solvents on drying-oil films: parts I and II. Journal of the Oil and Colour Chemists’ Association.

[25] Phenix A (2002). The Swelling of Artists’ Paints in Organic Solvents. Part 1, a Simple Method for Measuring the In-Plane Swelling of Unsupported Paint Films. Journal of the American Institute for Conservation.

[26] Phenix A (2002). The Swelling of Artists’ Paints in Organic Solvents. Part 2, Comparative Swelling Powers of Selected Organic Solvents and Solvent Mixtures. Journal of the American Institute for Conservation.

[27] Baij L, Hermans JJ, Keune K, Iedema PD (2018). Time-Dependent ATR-FTIR Spectroscopic Studies on Solvent Diffusion and Film Swelling in Oil Paint Model Systems. Macromolecules.

[28] Masschelein-Kleiner, L. *Les solvants*. Cours de conservation (Institut royal du patrimoine artistique, Bruxelles, 1994).

[29] Prati S (2019). Cleaning oil paintings: NMR relaxometry and SPME to evaluate the effects of green solvents and innovative green gels. New Journal of Chemistry.

[30] Blümich B (1998). The NMR-MOUSE: Construction, excitation, and applications. Magnetic Resonance Imaging.

[31] Angelova LV, Ormsby B, Richardson E (2016). Diffusion of water from a range of conservation treatment gels into paint films studied by unilateral NMR. Microchemical Journal.

[32] Fercher A, Briers J (1981). Flow visualization by means of single-exposure speckle photography. Optics Communications.

[33] van der Kooij HM, Sprakel J (2015). Watching paint dry; more exciting than it seems. Soft Matter.

[34] van der Kooij HM, Fokkink R, van der Gucht J, Sprakel J (2016). Quantitative imaging of heterogeneous dynamics in drying and aging paints. Scientific Reports.

[35] Pérez A (2018). A Portable Dynamic Laser Speckle System for Sensing Long-Term Changes Caused by Treatments in Painting Conservation. Sensors.

[36] Buijs J, van der Gucht J, Sprakel J (2019). Fourier transforms for fast and quantitative Laser Speckle Imaging. Scientific Reports.

[37] Kühn, H. Zinc White. In Feller, R. (ed.) *Artist’s pigments: A handbook of their history and characteristics, vol. 1*, 169–186 (Cambridge University Press and National Gallery of Art, Cambridge and London, 1986).

[38] Baij L, Chassouant L, Hermans JJ, Keune K, Iedema PD (2019). The concentration and origins of carboxylic acid groups in oil paint. RSC Advances.

[39] Hageraats, S. *et al*. Synchrotron deep-UV photoluminescence imaging for the submicron analysis of chemically altered zinc white oil paints. *Analytical Chemistry* acs.analchem.9b02443, 10.1021/acs.analchem.9b02443 (2019). 10.1021/acs.analchem.9b02443 31660714

[40] Ankersmit, B. & Stappers, M. H. *Managing Indoor Climate Risks in Museums*. Cultural Heritage Science (Springer International Publishing, Cham, 2017).

[41] Zumbühl S (2014). Parametrization of the solvent action on modern artists’ paint systems. Studies in Conservation.

[42] Modugno F (2019). On the influence of relative humidity on the oxidation and hydrolysis of fresh and aged oil paints. Scientific Reports.

[43] Casadio, F. *et al*. *Metal Soaps in Art: Conservation and Research*. Cultural Heritage Science (Springer International Publishing, Cham, 2019).

[44] van der Wel G, Adan O (1999). Moisture in organic coatings - a review. Progress in Organic Coatings.

[45] Michalski S (1990). A Physical Model Of The Cleaning Of Oil Paint. Studies in Conservation.

[46] Hermans JJ, Keune K, van Loon A, Corkery RW, Iedema PD (2016). Ionomer-like structure in mature oil paint binding media. RSC Advances.

[47] Hermans JJ (2019). 2D-IR spectroscopy for oil paint conservation: Elucidating the water-sensitive structure of zinc carboxylate clusters in ionomers. Science Advances.

[48] Vergeer, M., van den Berg, K. J., van Oudheusden, S. & Stols-Witlox, M. Evolon CR microfibre cloth as a tool for varnish removal. *CMOP proceedings*, 10.1007/978-3-030-19254-9 (2019).

[49] Arecchi T (2006). A new tool for painting diagnostics: Optical coherence tomography. Optics and Spectroscopy.

[50] Phenix A (2013). Effects of organic solvents on artists’ oil paint films: swelling. Smithsonian Contributions to Museum Conservation.

[51] Prati S (2018). Sustainability in art conservation: a novel bio-based organogel for the cleaning of water sensitive works of art. Pure and Applied Chemistry.

[52] Domingues JAL (2013). Innovative Hydrogels Based on Semi-Interpenetrating p(HEMA)/PVP Networks for the Cleaning of Water-Sensitive Cultural Heritage Artifacts. Langmuir.

[53] Fife, G., Och, J. V., Stabik, B., Miedema, N. & Seymour, K. Keywords: paintings, varnish removal, gel impregnated tissues, technique development. *ICOM-CC 16th triennial conference Lisbon 19-23 September 2011: preprints* (2011).

