# Supplementary material for: Quantifying solvent action in oil paint using portable laser speckle imaging
Source: Sci Rep. 2020 Jun 29;10:10574. doi: 10.1038/s41598-020-67115-1 (PMC7324590; doi:10.1038/s41598-020-67115-1)
Supplement: Supplementary file 1 — Supplementary information [file 41598_2020_67115_MOESM1_ESM.pdf]

Supporting Information for:

Quantifying solvent action in oil paint using portable laser speckle imaging

Lambert Baij,[*a,b] Jesse Buijs,[*c] Joen J. Hermans,[a,b] Laura Raven,[b]
Piet D. Iedema[a], Katrien Keune[a,b] and Joris Sprakel[c]

[*] *E-mail: C.L.M.Baij@uva.nl* [*] *E-mail: Jesse.Buijs@wur.nl*

[a] *Van 't Hoff Institute for Molecular Sciences, University of Amsterdam, PO box 94720, 1090GD Amsterdam, The Netherlands.*

[b] *Rijksmuseum Amsterdam, Conservation and Science, PO box 74888, 1070DN Amsterdam, The Netherlands.*

[c] *Wageningen University and Research, Department of Physical Chemistry and Soft Matter, Wageningen, The Netherlands*

List of Figures

1 Triplo Evolon loaded with 51% ethanol 2
2 The effect of Varnish on the LSI signal 3
3 Choice of ROI for integration of FT-LSI signal 4
4 OCT analysis of varnished model paints 5
5 Fitting results of the power spectra of the artificially aged model paint systems . 7

Triplo Evolon loaded with 51% ethanol

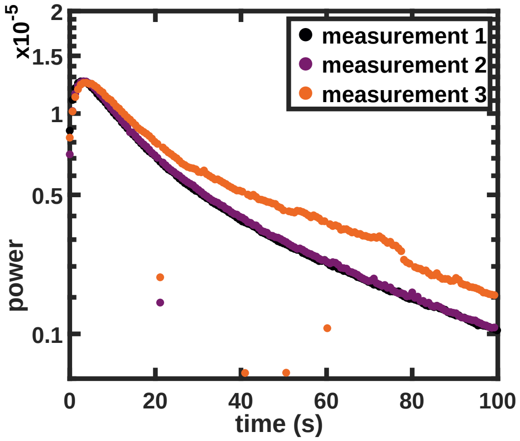

Figure 1 FT-LSI signal decay for a triplicate measurement using Evolon loaded with 51% ethanol on unvarnished paint. The black and purple series are nearly identical while the orange series measures a slightly higher activity. This deviation is probably caused by inaccurate application of solvent rather than inaccuracy of the measurement. This means that measurements are very sensitive to small deviations in the preparation. However the measured difference is small compared to measured trends in this article.

The effect of Varnish on the LSI signal

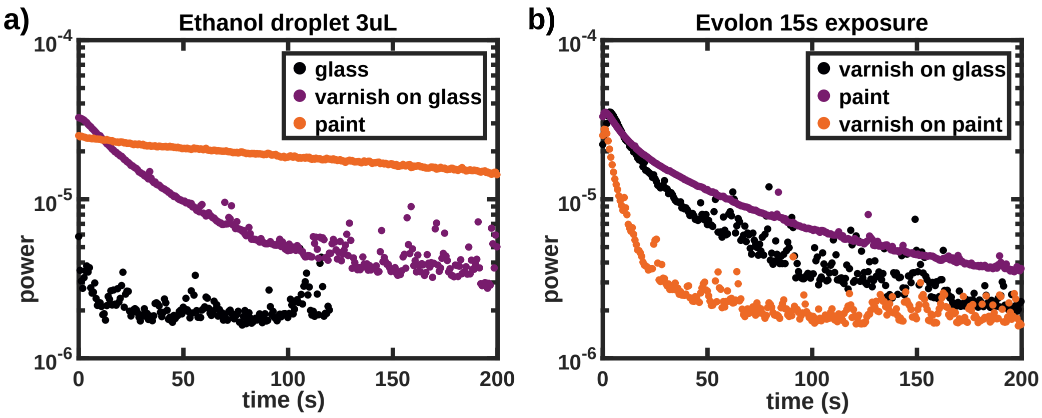

Figure 2 a: FT-LSI signal decay (1.6 Hz) for a 1 minute exposure of a drop of ethanol on glass, varnish and paint separately. Paint and varnish have the same signal initially, but the signal for varnish decays much faster. The wetted glass gives a baseline signal. b: FT-LSI signal decay (1.6 Hz) for a 15s evolon exposure, after which a varnish layer is not completely removed. The varnish signal is not neglegible compared to the paint signal. However, in the measurements discussed in the main text it was judged with a UV lamp that there was no varnish left.

Choice of ROI for integration of FT-LSI signal

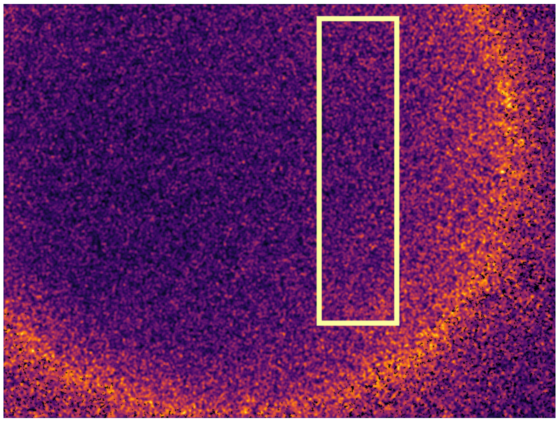

Figure 3 Example of ROI for integration and computation of FT-LSI signal decay. In all cases, the ROI was chosen in such a way the the region was as large as possible while still spatially homogeneous drying is observed (every pixel in the square dries at approximately the same rate).

OCT analysis of varnished model paints

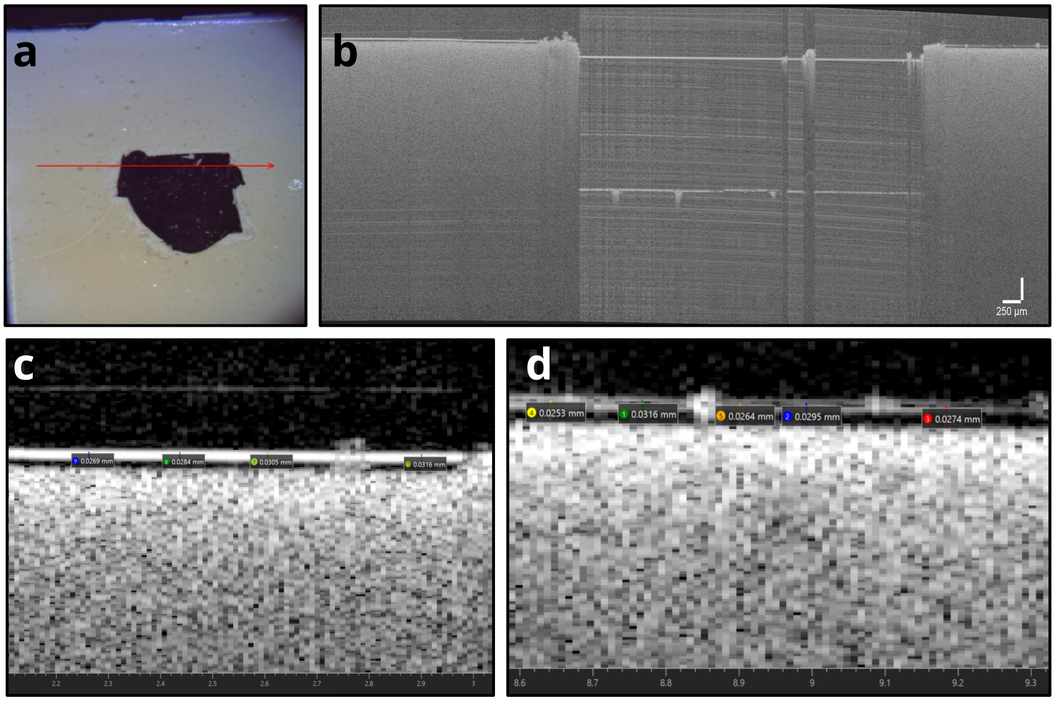

Figure 4 a: Area used for OCT scanning. **b**: overview OCT image, **c** and **d**: zoomed in examples of thickness determination, average thickness 0.03 mm, standard deviation 0.002 mm.

Varnish thicknesses of LO-ZnO samples

Table 1 Varnish thicknesses of LO-ZnO samples with 1–4 varnish layers. A constant (1:1 wt.) pigment to oil ratio was used. Samples were varnished with a dammar solution in shellsol T using a brush and subsequently aged for 7 day under UV-A and UV-B radiation. The total radiation dosage was $1{,}4 \cdot 10^{7}$ J/cm^{2} (UV-A) and $5{,}2 \cdot 10^{7}$ J/cm^{2} (UV-B). The average refractive index (ri) of dammar was determined to be 1.425.

**Sample**	Thickness (ri=1.0)	Thickness (ri=1.46)
LO-ZnO-1layer	30 μm	21 μm
LO-ZnO-2layer	20 μm	14 μm
LO-ZnO-3layer	13 μm	9 μm
LO-ZnO-4layer	<10 μm	<7 μm

Fitting of the power spectra to obtain α

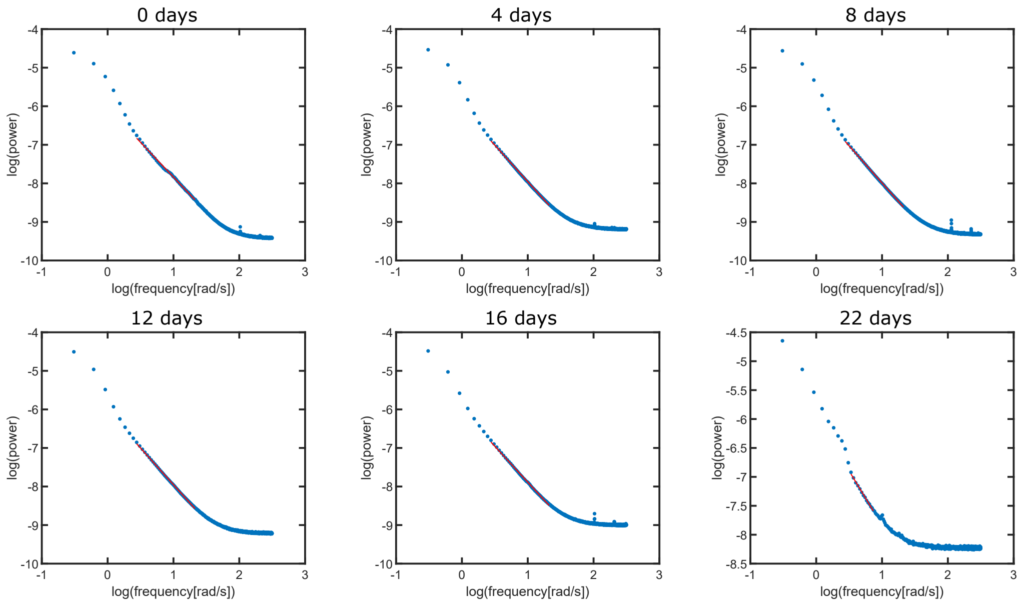

Figure 5 The power spectra (blue dots) belonging to the experiments shown in Figure 3 (main text) are fitted with a linear function on a log-log scale. The fitting result (red line) for each of the six samples is shown with the accelerated ageing time in the title of each sub-figure. From the slope of this fit the parameter α (Figure 3c, main text) is calculated, which gives information over the nature of the measured motion (convective vs diffusive).

ZnO-LO-22d.avi: Spreading of solvent through a cracked paint

Movie ZnO-LO-22d.avi shows spatial information obtained from a LSI experiment with a drop of ethanol on the ZnO-LO-22d cracked paint. The left half shows the raw speckle images which are shown exactly how they are obtained from the LSI camera. The right half shows analysed FT-LSI images where the magnitude of the 1.6 Hz frequency is visualised with a colour-map, where lighter colours correspond to higher dynamics. Both movies are played in parallel at 4x speed. Key moments in the movie are: application of the solvent (0 s), start of droplet area shrinking due to evaporation (4 s), disappearance of liquid on top of paint due to swelling and evaporation (10 s), liquid has disappeared everywhere except in the cracks (22 s). The movie has been compressed to facilitate online accessibility (2 times x-compression, 2 times y-compression and 4x frame-rate reduction), the original video is available on request.

ZnO-LO-0–16d.avi: Drop of ethanol on a young paint

Movies ZnO-LO-0–16d.avi show spatial information obtained from LSI experiments with a drop of ethanol on ZnO-LO-0–16d paint. The movies show analysed FT-LSI images where the magnitude of the 1.6 Hz frequency is visualised with a colour-map, where lighter colours correspond to higher dynamics. The movies are played at 4x speed. The movies have been compressed to facilitate online accessibility (2 times x-compression, 2 times y-compression and 2x frame-rate reduction), the original videos are available on request.
